# Quantification and Trends of Antimicrobial Use in Commercial Broiler Chicken Production in Pakistan

**DOI:** 10.3390/antibiotics10050598

**Published:** 2021-05-18

**Authors:** Muhammad Umair, Muhammad Farooq Tahir, Riasat Wasee Ullah, Jabir Ali, Naila Siddique, Ayesha Rasheed, Muhammad Akram, Muhammad Usman Zaheer, Mashkoor Mohsin

**Affiliations:** 1 Institute of Microbiology, University of Agriculture, Faisalabad 38000, Pakistan; drmumair13@gmail.com (M.U.); jabirjinnah@gmail.com (J.A.); 2 The Fleming Fund Country Grant Pakistan, Health Security Partners, Washington, DC 20037, USA; drmftahir@gmail.com (M.F.T.); muhammad-usman.zaheer@fulbrightmail.org (M.U.Z.); 3 Livestock Wing, Ministry of National Food Security and Research, Islamabad 44000, Pakistan; riasatwasee@gmail.com (R.W.U.); muhammadakram422@yahoo.com (M.A.); 4 National Reference Laboratory for Poultry Diseases, Animal Sciences Institute, National Agriculture Research Center, Islamabad 44000, Pakistan; naila.nrlpd@gmail.com; 5 The Fleming Fund Country Grant Pakistan, DAI, Islamabad 44000, Pakistan; ayesha.rasheed@gmail.com

**Keywords:** antimicrobial use, broiler chicken, surveillance, critically important antimicrobials, Pakistan

## Abstract

Antimicrobial resistance (AMR) is a global health challenge and antimicrobial use (AMU) in the livestock sector has been considered as one of the contributing factors towards the development of AMR in bacteria. This study summarizes the results of a point prevalence survey conducted to monitor farm-level AMU in commercial broiler chicken farms in Punjab and Khyber Pakhtunkhwa (KPK) provinces of Pakistan. A cross-sectional study was conducted to quantify AMU and to check seasonal variations of AMU in 12 commercial broiler chicken farms (six from each province) during the summer and winter seasons of the year 2020–2021. AMU was recorded using three AMU metrics: kg, mg per population correction unit (mg/PCU), and mg/kg of final flock weight. A total of 22 antimicrobial drugs (348.59 kg) were used for therapeutic or prophylactic purposes in surveyed broiler chicken farms. The total combined AMU for all the broiler chicken farms was 462.57 mg/PCU. The use of most of the antimicrobials increased during winter flocks compared to summer. The top three antimicrobial drugs used during the summer were neomycin (111.39 mg/PCU), doxycycline (91.91 mg/PCU), and tilmicosin (77.22 mg/PCU), whereas doxycycline (196.81 mg/PCU), neomycin (136.74 mg/PCU), and amoxicillin (115.04 mg/PCU) during the winter. Overall, 60% of the antibiotics used in broiler chicken were critically important antimicrobial classes (CIA) for human medicine as characterized by the World Health Organization. Our findings showed high AMU in broiler chicken production and a call for urgent actions to regulate CIA use in food animals in Pakistan. This baseline survey is critical for the design and implementation of a subsequent national level AMU surveys that can include additional farming types, animals’ species, and geographical locations over a longer period of time.

## 1. Introduction

A political commitment to combat AMR has increased significantly since the 68th World Health Assembly approved the Global Action Plan (GAP) on AMR in 2015 [1]. Surveillance of Antimicrobial Use (AMU) in food animals and its reduction in animal husbandry is one of the key strategic objectives indicated in many regional and global initiatives to address the AMR crisis. This includes the GAP and subsequent plans developed by the Food and Agriculture Organization of the United Nations (FAO) and World Organisation for Animal Health (OIE) [2,3]. Consequently, many countries, including Pakistan, have drafted their National Action Plans based on the “One Health” approach to tackle the AMR problem. This requires AMU surveillance in food animals to address critical knowledge gaps and to promote the rational use of antimicrobials and implement antimicrobial stewardship programs in food animals production [4].

AMU surveillance in food animals is crucial to address the growing challenge of AMR. Demand for animal-source nutrition is rising globally and is driving the growth of intensive livestock farming practices where antimicrobials are used not only for treatment against infectious diseases, but also for prophylactic and growth promotion purposes. Antimicrobial usage in agriculture selects resistant bacteria and genetic determinant which may be transmitted to humans through direct contact with animals, the food chain, or the environment. The major public health significances of antimicrobial residues in the food chain include the development of drug resistance in bacteria, drug hypersensitivity reaction, disruption of normal intestinal flora and carcinogenic effects [5]. Global AMU data suggest that the use of antimicrobials in animal production sectors is much larger than its consumption in human medicine [6]. Recently, van Boeckel et al. have estimated global antimicrobial sales to be 93,309 tonnes in 2017 and expect an increase of 11.5% to 104,079 tonnes by 2030 [7]. Despite the growing recognition of the urgency to tackle AMR in many low- and middle-income countries (LMICs), there is a lack of data on the trends and quantities of AMU in food animals both at the country level and farm level [8].

The National Action Plan on AMR has been developed as a comprehensive document that describes Pakistan’s vision for AMR prevention and control. The plan calls for immediate reduction in AMU and level of AMR in human and animal sectors. However, data on trends and quantities of AMU in food animals in Pakistan is very limited, presenting challenges in implementing evidence-based policies and programmes. Mohsin et al. have published the first quantitative analysis of AMU in commercial poultry in Pakistan [9]. The survey was conducted at a single commercial broiler chicken production facility over a period of 5 years and estimated the farm level AMU to be as high as 250 mg/kg of the final flock weight. This figure surpasses the volume of antimicrobials used per kilogram of chicken in different countries except China [10]. The estimation and reduction of AMU in animal health sector is the central goal of Pakistan’s National Action Plan on AMR. The inappropriate use of antimicrobials in commercial chicken production is a primary concern. The estimation of AMU in intensive livestock farming is essential to devise and implement antimicrobial stewardship programs. However, there is a gap in the knowledge of use of antimicrobials and effects of seasonal variations in broiler chicken production in Pakistan. This study aimed to assess (1) the quantitates of antimicrobial use; (2) the effect of seasonal variations on AMU; (3) and the use of critically important antimicrobial classes in commercial broiler chicken production in Pakistan.

## 2. Material and Methods

### 2.1. Study Design

A cross sectional survey was conducted to collect the antimicrobial use data in commercial broiler chicken farms in summer (August to September 2020) and winter (November to January 2021). The farms were selected from Punjab and Khyber Pakhtunkhwa (KPK) provinces as these provinces have a majority of the poultry farms in Pakistan. Poultry distribution is very sporadic in Pakistan and small-scale farmers hardly maintain any production records therefore, keeping in view this issue only medium to large-scale commercial farms were selected. Commercial broiler chicken farms are mainly centered in Punjab and KPK only.

### 2.2. Selection Criteria

**Figure 1:**
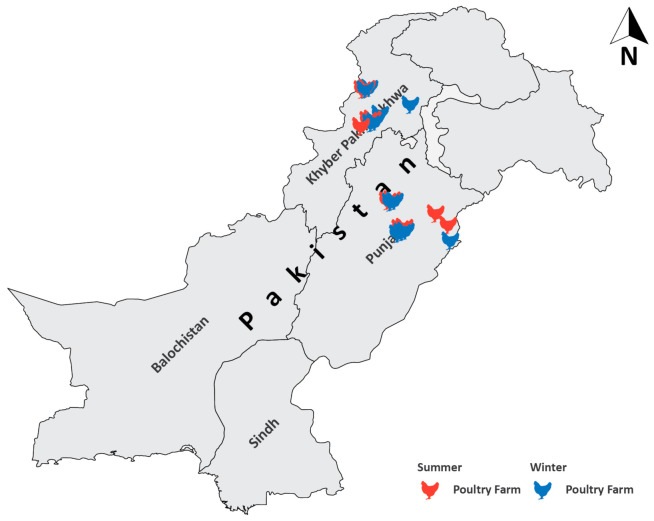
Geographical locations of broiler chicken farms in Punjab and Khyber Pakhtunkhwa.

A total of 12 commercial broiler chicken farms rearing more than 2000 birds were selected for AMU data collection. All the summer farms were contacted again for winter data collection. The farms, which did not agree to participate again, were replaced with new farms as per the selection criteria mentioned above. A list of nominated farms along with their locations is given in Table 1 and Figure 1.

### 2.3. Data Collection

Data were collected using a pre-tested survey questionnaire/data collection tool (DCT). The persons involved in data collection were trained for the data collection during a series of on-site trainings. To minimize the error, trash cans were placed at each farm and the farm supervisors were asked to discard the empty antibiotics vials/bottles in those cans. The data collection process was monitored virtually as well as random on-site inspections. The data were collected for one production cycle for broiler chicken (35–42 days) in winter and summer. At the end of the survey, the DCT and empty vials were collected from the farms for data compilation and analysis.

### 2.4. Antimicrobial Use Calculations

AMU was calculated using the following three metrics:

#### 2.4.1. Antimicrobial Active Ingredient (AAI)

Formulation of each antimicrobial product, i.e., antimicrobial active ingredient/s and their concentration/s were taken from the trash can contents or online searching the respective product. Product quantities used and AAI concentrations were employed to calculate the amount of active ingredient used for each antimicrobial (Equation (1)).

(1) AAIkg=Amount of product usedg−ml×Conc. of antimicrobialmgg−ml1,000,000

#### 2.4.2. Milligrams of AAI Used per Population Correction Unit (mg/PCU)

The total amount of AAI used in milligrams was divided by the population correction unit (PCU) of the flocks on which the respective antimicrobial is administered to calculate mg/PCU. PCU was calculated by multiplying the number of birds in respective flocks with 1 kg, the standardized average weight of broiler chicken at the time of treatment, as defined by the European Surveillance of Veterinary Antimicrobial Consumption (ESVAC) [11] (Equations (2) and (3)).

(2) $$mg/PCU_{mg/kg}=\frac{Total\;AAI_{mg}}{Population\;of\;treated\;flocks\times 1\;kg}$$

The cumulative mg/PCU was calculated as:

(3) $$\overset{N}{\underset{n=1}{\sum }}mg/PCU_{mg/kg}=\frac{\sum_{n=1}^{N}{\left(Total\;AAI_{mg}\right)}_{N}}{\sum_{n=1}^{N}{\left(PCU\right)}_{N}}$$

where *N* is the total number of flocks treated.

#### 2.4.3. Milligrams of AAI Used per Final Flock Weight (mg/FFW)

The total amount of AAI used in milligrams was divided by the final flock weight (FFW) (weight at the time of harvesting) on which the respective antimicrobial is administered [9] (Equations (4) and (5)).

(4) $$mg/FFW_{mg/kg}=\frac{Total\;AAI_{mg}}{FFW}$$

The cumulative mg/FFW was calculated as:

(5) $$\overset{N}{\underset{n=1}{\sum }}mg/FFW_{mg/kg}=\frac{\sum_{n=1}^{N}{\left(Total\;AAI_{mg}\right)}_{N}}{\sum_{n=1}^{N}{\left(FFW\right)}_{N}}$$

where *N* is the total number of flocks treated.

### 2.5. Calculations for WHO Critically Important Antimicrobial Classes

All the antimicrobial drugs used during the broiler chicken production were also categorized according to the WHO list of critically important antimicrobial classes for human medicine (WHO-CIA) [12]. AAI percentages for different WHO-CIA categories were calculated to add a public health perspective to this study.

## 3. Results

### 3.1. Response to Antimicrobial Use Data Collection

Data collectors provided AMU data from all the 12 farms in winter and 11 out of 12 farms in summer, while the 12th farm dropped out. Two farms each from Punjab and KPK participated both in the summer and winter data collection, while the rest of farms were different for the two seasons. A total of 19 farms rearing 33 flocks were studied during the summer and winter. The list of broiler chicken farms, number of birds, and locations are mentioned in Table 1 and Figure 1.

### 3.2. Population Size and Housing

The broiler chicken farms in this study belonged to the 10 different districts from Pakistan. The farms reared a total of 765,500 birds with 96% reared in environmentally controlled sheds and the remaining 4% in conventional housing. Farms practicing conventional housing belonged to KPK rearing ≤7500 birds (Table 1; Figure 1). An average production cycle of 38 days was observed in this study.

### 3.3. Quantitaive Antimicrobial Use in Broiler Chicken

**Figure 2:**
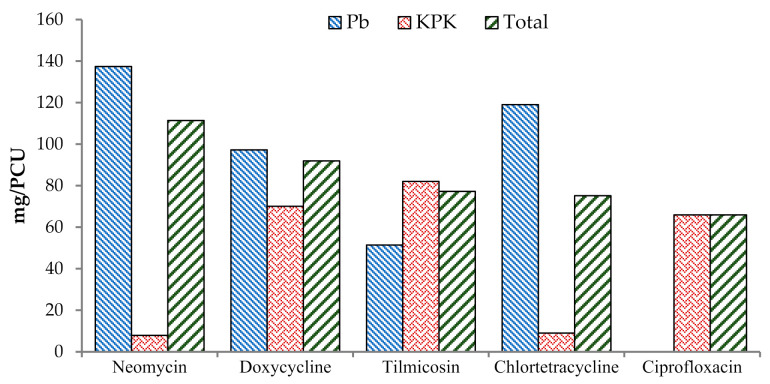
Top five antimicrobial agents (mg/PCU) in broiler chicken during summer.

**Figure 3:**
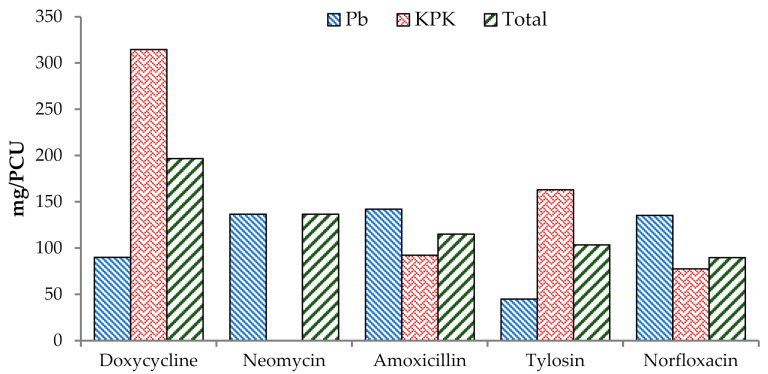
Top five antimicrobial agents (mg/PCU) in broiler chicken during winter.

A total of 22 antimicrobials drugs (348.59 kg) were used for therapeutic or prophylactic purposes on surveyed broiler chicken farms (Table 4). None of the participating farms used antimicrobials as a feed premix. The total combined AMU was 222.55 mg/kg of final flock weight and 462.57 mg/PCU (Table 4). The top three antimicrobial drugs used during the summer were neomycin (111.39 mg/PCU), doxycycline (91.91 mg/PCU), and tilmicosin (77.22 mg/PCU) (Table 2; Figure 2), whereas doxycycline (196.81 mg/PCU), neomycin (136.74 mg/PCU), and amoxicillin (115.04 mg/PCU) during the winter (Table 3, Figure 3).

### 3.4. Seasonal Variations in Antimicrobial Use in Broiler Chicken

**Figure 4:**
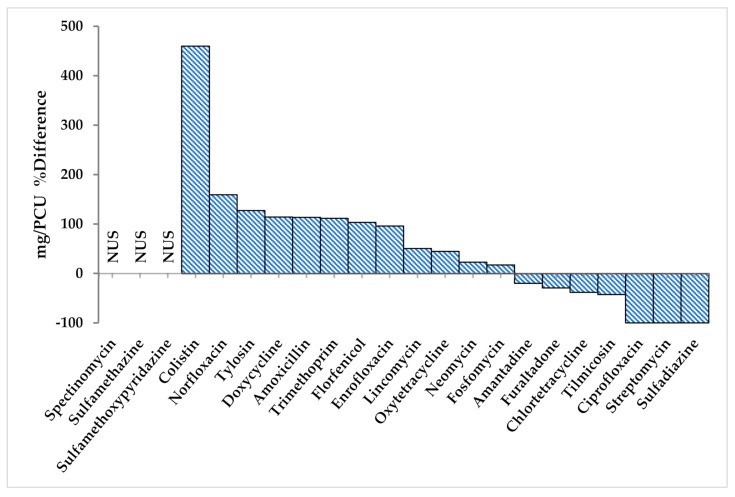
The AMU % difference in winter as compared to summer values in broiler chicken. NUS: Not used in summer.

Results from our study indicated that in broiler chicken, the overall AMU increased (117%) in winter flocks for most of the antimicrobials including fosfomycin, neomycin, oxytetracycline, lincomycin, enrofloxacin, florfenicol, trimethoprim, amoxicillin, doxycycline, tylosin, norfloxacin, colistin, and spectinomycin. An increase was more pronounced for colistin in winter (459.7%) when compared to summer (12.01 to 67.22 mg/PCU) (Figure 4; Table 4).

### 3.5. Use of WHO-CIA in Broiler Chicken

**Figure 5:**
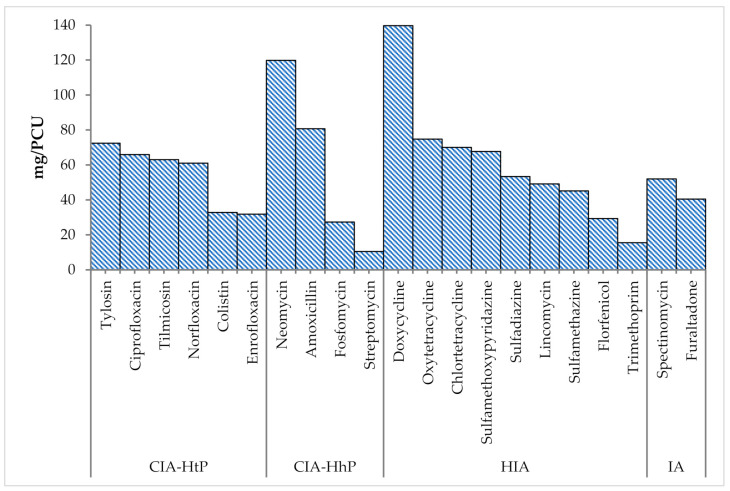
Use of critically important antimicrobial classes (mg/PCU) in broiler chicken.

Overall, 60% of antimicrobials used in broiler chicken fall within the category of critically important antimicrobial classes (CIA) for human medicine, as defined by the WHO. Figure 5 provides details on the use of CIA with highest priority (CIA-HtP), CIA with high priority (CIA-HhP), highly important antimicrobials (HIA), and important antimicrobials (IA) in broiler chicken.

## 4. Discussion

The AMU surveillance in food animal production systems is one of the key objectives of the Global Action Plan on AMR by WHO [1]. In many European Union countries, the implementation of national and farm-level AMU surveillance programmes has resulted in a substantial reduction of AMU in food animals [11]. There are, however, a few national level studies on AMU in food animals from LMICs. Pakistan lacks any formal AMU surveillance in food animals due to many reasons but weak legislation and poor implementation of the any existing legislation are among the leading causes. Punjab has recently enforced the Punjab Animals Feed Stuff and Compound Feed Act (2016) and the Punjab Poultry Production Act (2016) which provide a legal cover for monitoring the animal feed production for good husbandry practices including antimicrobial use.

The current study provides quantitative AMU data in support of the on-going national AMR surveillance efforts in Pakistan and is the first study conducted on a relatively large geographical area, i.e., representing commercial broiler chicken production from two provinces of Pakistan. To the best of our knowledge, none of the AMU studies from South Asia cover such a large cohort and geographical area. The absence of internationally accepted standardized AMU indicators also present a difficulty in comparison of data across species, production type, and countries [13]. In the present study, we used multiple AMU indicators for broiler chicken, i.e., kg, mg/PCU, and mg/kg of final flock weight. The use of multiple AMU indicators is valuable and provides more comprehensive AMU data.

Our results demonstrate a very high AMU in commercial broiler chicken farms (462.5 mg/PCU) compared to two previous farm-level studies from Canada of 134 [14] and 98–104 mg/PCU [15]. This figure is also excessively high when compared to the sales-data based global AMU of 148 mg/PCU [6]. However, this amount is comparatively low from a previous study in broiler chicken from Pakistan where AMU was 251 mg/kg approximately 502 mg/PCU [9] but higher than reported from Morocco (63.48 mg/kg of the average weight at treatment) [16]. Differences in production types and calculation methodologies pose difficulties in comparing data among different countries and regions. European countries mainly publish their sales data using various methodologies. Different reports from Europe publishing AMU data in food animals include ANSES-ANMV (France), BelVet-SAC (Belgium), DANMAP (Denmark), NethMap (Netherlands), SWEDRES-SVARM (Sweden), and UK-VARSS (UK) [17,18,19,20,21,22]. ESVAC also publish a yearly sales data from different European countries [11]. Comparing sales data from the UK (12 mg/kg in broilers) and France (34.24 mg/kg in poultry) our results show substantially high amounts of antimicrobials being used in broiler chicken production.

All the surveyed broiler chicken farms were observed administering prophylactic antimicrobial courses at different stages of the production cycle. In particular, all the farms administered prophylactic antimicrobial courses during the first week of a bird’s life. Oral antimicrobial administration via drinking water was the only source of antimicrobials in this study. Farmers were unaware of any antimicrobials pre-added in a commercial feed. The use of antimicrobials for prophylactic courses in a routine practice, could be avoided by the introduction of good hygiene and management practices at farms.

During the studied period in summer (Aug–Sep), the average maximum temperatures recorded in Punjab and KPK were 34.5 and 32.5 °C, whereas during winter (Nov-Jan), the average minimum temperatures were 7 and 4 °C, respectively [23]. As 94% of the birds were reared in environmentally controlled sheds, the environment temperature variations do not affect much in terms of stress. The high AMU during winter observed in this study may be linked to the compromised ventilation during winter resulting in high humidity, poor litter condition, and accumulation of obnoxious gases [24]. Shoaib et al. in 2019 observed a high incidence of certain bacterial infections during winter as compared to the summer season [25]. Moreover, transportation stress on chicks from hatchery to sheds during winter is likely to be associated with the high prophylactic treatments during the first week of winter production cycle [26].

The overall high AMU during winter observed in this study is in agreement with the study from Morocco where a high frequency of treatments was noted during winter [16]. Similarly, high values for tylosin during winter are parallel to the observation made by Agunos et al. from Canada [27].

The percentage use of critically important antimicrobial classes (60%) in our study was found comparable to the studies from other countries such as Belgium (61%) [28], Thailand (63%) [29], and Europe (76%) [30], but higher than from Vietnam (36.4%) [31]. The use of CIA in veterinary medicine requires strict regulations in Pakistan.

Our data represent the overall 33 flocks of broiler chicken across two provinces and account for the summer and winter season. However, a longitudinal study with the inclusion of other farms and the farming systems is required for sustainable monitoring to understand AMU. In some cases, volumes of antimicrobial products used were not provided. Therefore, the usage was calculated based upon daily water intake from respective breed performance manuals [9]. Caution is necessary in the interpretation of the results.

One of the main limitations of our study is that it was based on a convenient sample of broiler chicken farms that are not representative of the country’s commercial poultry which includes open housed chicken and layers. Our results are therefore not generalizable to the rest of Pakistan’s poultry sector.

The current study highlights the need for a robust and sustainable AMU surveillance and monitoring strategy for food animals in Pakistan. In the future, AMU in food animals should be strongly regulated to reduce the risk of AMR development.

## 5. Conclusions

Antimicrobials are being excessively used as prophylactic or therapeutic measures in broiler chicken production. A large percentage of the on-farm AMU is of critically important antimicrobial classes with the highest or high priority for human medicine. The antimicrobial use in winter flocks was found considerably high when compared to the summer data. These findings should provide policymakers with high-resolution AMU data at the farm-level to devise national-level strategies to monitor AMU in food animals and to combat the AMR crisis.

## Figures and Tables

**Table 1:** Commercial broiler chicken farms’ locations and number of birds.

Punjab			KPK		
**Summer**					
**Farm ID**	**Location** **(District)**	**No. of** **Birds**	**Farm ID**	**Location** **(District)**	**No. of** **Birds**
PP-1	Faisalabad	26,500	KP-1	Mardan	56,000
PP-2	Sargodha	30,000	KP-2	Mardan	52,000
PP-3	Sargodha	30,000	KP-3	Peshawar	108,000
PP-4	Faisalabad	19,900	KP-4	Lower Dir	6000
PP-5	Lahore	64,600	KP-5	Lower Dir	3000
PP-6	Sheikhupura	81,600	--	--	--
**Winter**					
PP-1	Faisalabad	24,000	KP-1	Mardan	58,000
PP-4	Faisalabad	15,000	KP-2	Mardan	56,000
PP-7	Sargodha	28,500	KP-6	Lower Dir	6800
PP-8	Sargodha	29,000	KP-7	Lower Dir	7500
PP-9	Faisalabad	26,000	KP-8	Swabi	3500
PP-10	Kasur	30,600	KP-9	Mansehra	3000

**Table 2:** Antimicrobial use in broiler chicken during summer.

Antimicrobial Class	Antimicrobial	AAI		mg/PCU		mg/FFW		Total		
		Pb	KPK	Pb	KPK	Pb	KPK	AAI	mg/PCU	mg/FFW
Antiviral	Amantadine	2.21	0	22.11	0	10.21	0	2.21	22.11	10.21
Aminopenicillins	Amoxicillin	6.69	9.52	78.59	44.18	36.35	21.91	16.2	53.92	26.2
Tetracyclines	Chlortetracycline	9.89	0.5	119	9.01	57.33	4.69	10.39	75.16	37.3
Quinolones and fluoroquinolones	Ciprofloxacin	0	3.63	0	65.9	0	34.27	3.63	65.9	34.27
Polymyxins	Colistin	3.89	1.75	15.65	7.91	7.33	3.94	5.64	12.01	5.78
Tetracyclines	Doxycycline	24.16	4.27	97.26	70.08	45.54	36.8	28.44	91.91	43.97
Quinolones and fluoroquinolones	Enrofloxacin	7.18	0.93	31.37	8.56	14.7	4.06	8.11	24	11.29
Amphenicols	Florfenicol	1.78	0.76	28.02	12.52	13.64	6.58	2.54	20.43	10.32
Phosphonic acid derivatives	Fosfomycin	0	0.07	0	23.93	0	14.53	0.07	23.93	14.53
Nitrofurans derivatives	Furaltadone	0.96	0	49.22	0	22.93	0	0.96	49.22	22.93
Lincosamides	Lincomycin	2.2	0.03	37.28	10.46	18.35	6.35	2.23	36	17.89
Aminoglycosides	Neomycin	30.09	0.43	137.43	7.89	63.89	4.1	30.52	111.39	52.91
Quinolones and fluoroquinolones	Norfloxacin	1.24	1.42	63.15	24.85	29.42	13.06	2.65	34.64	17.62
Tetracyclines	Oxytetracycline	3.3	0	59.38	0	26.55	0	3.3	59.38	26.55
Aminoglycosides	Streptomycin	0	0.03	0	10.46	0	6.35	0.03	10.46	6.35
Sulfonamides	Sulfadiazine	4.28	0	53.36	0	24.6	0	4.28	53.36	24.6
Macrolides and ketolides	Tilmicosin	1.52	12.91	51.43	82.06	25.44	39.91	14.43	77.22	37.65
Dihydrofolate reductase inhibitors	Trimethoprim	0.86	0	10.67	0	4.92	0	0.86	10.67	4.92
Macrolides and ketolides	Tylosin	12.08	2.15	48.63	33.7	22.77	17.81	14.24	45.58	21.85
	Total	112.32	38.42	452.11	173.6	211.69	86.41	150.74	320.9	154.58

AAI: Antimicrobial active ingredient; mg/FFW: Milligrams per final flock weight; mg/PCU: Milligrams per population unit; Pb: Punjab; KPK: Khyber Pakhtunkhwa. Units: AI (kg); mg/FFW (mg/kg); mg/PCU (mg/kg).

**Table 3:** Antimicrobial use in broiler chicken during winter.

Antimicrobial Class	Antimicrobial	AAI		mg/PCU		mg/FFW		Total		
		Pb	KPK	Pb	KPK	Pb	KPK	AAI	mg/PCU	mg/FFW
Antiviral	Amantadine	0	0.06	0	17.65	0	9.83	0.06	17.65	9.83
Aminopenicillins	Amoxicillin	15.2	11.74	142.01	92.3	58.79	51.78	26.94	115.04	55.53
Tetracyclines	Chlortetracycline	1.4	0	46.4	0	19.95	0	1.4	46.4	19.95
Polymyxins	Colistin	3.24	15.84	21.57	118.65	9.19	66.51	19.08	67.22	32.28
Tetracyclines	Doxycycline	12.2	38.7	89.98	314.52	38.28	176.51	50.9	196.81	94.61
Quinolones and fluoroquinolones	Enrofloxacin	4	4.18	37.95	60.98	16.09	38.7	8.18	47.02	22.94
Amphenicols	Florfenicol	3.54	0.23	42.49	30.69	17.03	17.04	3.77	41.5	17.03
Phosphonic acid derivatives	Fosfomycin	0	0.4	0	27.99	0	15.96	0.4	27.99	15.96
Nitrofurans derivatives	Furaltadone	1.05	0	34.8	0	14.96	0	1.05	34.8	14.96
Lincosamides	Lincomycin	4.93	3.78	46.75	68.3	19.83	44.55	8.71	54.16	26.12
Aminoglycosides	Neomycin	18.54	0	136.74	0	58.17	0	18.54	136.74	58.17
Quinolones and fluoroquinolones	Norfloxacin	2	4.3	135.36	77.62	58.36	50.62	6.3	89.78	52.85
Tetracyclines	Oxytetracycline	6.6	0	85.87	0	35.04	0	6.6	85.87	35.04
Sulfonamides	Sulfamethazine	0	2.5	0	45.13	0	29.43	2.5	45.13	29.43
Sulfonamides	Sulfamethoxypyridazine	0	3.75	0	67.69	0	44.15	3.75	67.69	44.15
Aminocyclitols	Spectinomycin	0.53	3.78	19.2	68.3	6.55	44.55	4.31	51.99	26.04
Macrolides and ketolides	Tilmicosin	1.5	4.75	52.54	42.09	24.92	23.56	6.25	44.2	23.87
Dihydrofolate reductase inhibitors	Trimethoprim	0	1.25	0	22.56	0	14.72	1.25	22.56	14.72
Macrolides and ketolides	Tylosin	6.1	21.76	44.99	163	19.14	91.37	27.86	103.54	50.03
	Total	80.83	117.02	936.65	1217.47	396.3	719.28	197.85	697.01	334.68

AAI: Antimicrobial active ingredient; mg/FFW: Milligrams per final flock weight; mg/PCU: Milligrams per population unit; Pb: Punjab; KPK: Khyber Pakhtunkhwa. Units: AI (kg); mg/FFW (mg/kg); mg/PCU (mg/kg).

**Table 4:** Total antimicrobial use in broiler chicken split in seasons (summer and winter) and provinces (Punjab and Khyber Pakhtunkhwa).

WHO-CIA Classes	Antimicrobial Class	Antimicrobial	Summer			Winter			mg/PCU % Diff *	Punjab			KPK			Total		
			AAI	mg/FFW	mg/PCU	AAI	mg/FFW	mg/PCU		AAI	mg/FFW	mg/PCU	AAI	mg/FFW	mg/PCU	AAI	mg/FFW	mg/PCU
-	Antiviral	Amantadine	2.21	10.21	22.11	0.06	9.83	17.65	−20.17	2.21	10.21	22.11	0.06	9.83	17.65	2.27	10.2	21.96
CIA-HhP	Aminopenicillins	Amoxicillin	16.2	26.2	53.92	26.94	55.53	115.04	113.35	21.89	49.46	113.92	21.26	32.16	62.05	43.14	39.1	80.69
HIA	Tetracyclines	Chlortetracycline	10.39	37.3	75.16	1.4	19.95	46.4	−38.27	11.29	46.52	99.66	0.5	4.69	9.01	11.79	33.81	70.01
CIA-HtP	Quinolones and fluoroquinolones	Ciprofloxacin	3.63	34.27	65.9	0	0	0	−100	0	0	0	3.63	34.27	65.9	3.63	34.27	65.9
CIA-HtP	Polymyxins	Colistin	5.64	5.78	12.01	19.08	32.28	67.22	459.7	7.13	8.07	17.88	17.59	25.77	49.58	24.72	15.78	32.8
HIA	Tetracyclines	Doxycycline	28.44	43.97	91.91	50.9	94.61	196.81	114.13	36.36	42.82	94.69	42.97	128.14	233.52	79.34	66.97	139.67
CIA-HtP	Quinolones and fluoroquinolones	Enrofloxacin	8.11	11.29	24	8.18	22.94	47.02	95.92	11.18	15.17	33.44	5.11	15.13	28.78	16.29	15.15	31.82
HIA	Amphenicols	Florfenicol	2.54	10.32	20.43	3.77	17.03	41.5	103.13	5.32	15.72	36.22	0.99	7.67	14.51	6.31	13.49	29.32
CIA-HhP	Phosphonic acid derivatives	Fosfomycin	0.07	14.53	23.93	0.4	15.96	27.99	16.97	0	0	0	0.47	15.73	27.3	0.47	15.73	27.3
IA	Nitrofurans derivatives	Furaltadone	0.96	22.93	49.22	1.05	14.96	34.8	−29.3	2.01	17.95	40.47	0	0	0	2.01	17.95	40.47
HIA	Lincosamides	Lincomycin	2.23	17.89	36	8.71	26.12	54.16	50.44	7.13	19.35	43.36	3.81	42.44	65.31	10.94	23.88	49.11
CIA-HhP	Aminoglycosides	Neomycin	30.52	52.91	111.39	18.54	58.17	136.74	22.76	48.63	61.58	137.16	0.43	4.1	7.89	49.06	54.78	119.78
CIA-HtP	Quinolones and fluoroquinolones	Norfloxacin	2.65	17.62	34.64	6.3	52.85	89.78	159.18	3.24	42.42	94.21	5.72	29.55	50.85	8.95	33.19	61
HIA	Tetracyclines	Oxytetracycline	3.3	26.55	59.38	6.6	35.04	85.87	44.61	9.9	31.66	74.75	0	0	0	9.9	31.66	74.75
IA	Aminocyclitols	Spectinomycin	0	0	0	4.31	26.04	51.99	NUS	0.53	6.58	19.27	3.78	44.5	68.23	4.31	26.04	51.99
CIA-HhP	Aminoglycosides	Streptomycin	0.03	6.35	10.46	0	0	0	−100	0	0	0	0.03	6.35	10.46	0.03	6.35	10.46
HIA	Sulfonamides	Sulfadiazine	4.28	24.6	53.36	0	0	0	−100	4.28	24.6	53.36	0	0	0	4.28	24.6	53.36
HIA	Sulfonamides	Sulfamethazine	0	0	0	2.5	29.43	45.13	NUS	0	0	0	2.5	29.43	45.13	2.5	29.43	45.13
HIA	Sulfonamides	Sulfamethoxypyridazine	0	0	0	3.75	44.15	67.69	NUS	0	0	0	3.75	44.15	67.69	3.75	44.15	67.69
CIA-HtP	Macrolides and ketolides	Tilmicosin	14.43	37.65	77.22	6.25	23.87	44.2	−42.76	3.02	25.18	51.98	17.66	33.63	65.37	20.68	32.06	63
HIA	Dihydrofolate reductase inhibitors	Trimethoprim	0.86	4.92	10.67	1.25	14.72	22.56	111.43	0.86	4.92	10.67	1.25	14.72	22.56	2.11	8.13	15.53
CIA-HtP	Macrolides and ketolides	Tylosin	14.24	21.85	45.58	27.86	50.03	103.54	127.16	18.18	21.41	47.35	23.91	66.59	121.13	42.1	34.83	72.4
		Total	150.74	154.58	320.9	197.85	334.68	697.01	117.2	193.15	218.6	484.33	155.44	227.67	438.1	348.59	222.55	462.57

AAI: Antimicrobial active ingredient; mg/FFW: Milligrams per final flock weight; mg/PCU: Milligrams per population unit; NUS: Not used in summer, KPK: Khyber Pakhtunkhwa. Units: AI (kg); mg/FFW (mg/kg); mg/PCU (mg/kg). * % difference in winter AMU compared to the summer values.
